# Understanding Disparities in Breast Reconstruction Rates in Regional Populations Following Oncologic Resection of Breast Cancer: A 10‐Year Retrospective Observational Cohort Study

**DOI:** 10.1155/tbj/1281318

**Published:** 2026-03-08

**Authors:** Samuel M. Jansson, Calyb J. Austin, Mingchun Liu, Steven J. Craig

**Affiliations:** ^1^ Department of Surgery, The Wollongong Hospital, Wollongong, New South Wales, Australia; ^2^ Graduate School of Medicine, Faculty of Science Medicine and Health, University of Wollongong, Wollongong, New South Wales, Australia, uow.edu.au

## Abstract

**Introduction:**

Breast cancer is the most common cancer among women globally, with 2.3 million new cases in 2020. Globally, incidence rates of breast cancer are highest in Australia, yet only 29% of Australian women opt for breast reconstruction (BR). The decision‐making process for BR is complex, involving various surgical and nonsurgical considerations. Approximately 50% of women would choose BR if adequately informed and given the option. This study evaluates the factors contributing to reduced BR rates in regional and rural New South Wales over a 10‐year period.

**Methods:**

A multicentre, retrospective observational cohort study analysed 2052 women who were diagnosed with breast cancer in the Illawarra Shoalhaven Local Health District between 2012 and 2022, focussing on primary resection outcomes and other objective factors that contributed to BR rates. Descriptive statistics, chi‐squared tests and logistic regression were used to assess relationships between age, comorbidities, language and rurality, with reconstruction rates.

**Results:**

Among the 2052 women diagnosed with breast cancer who required oncologic resection, the mean age was 65 years. Only 127 (6.2%) underwent BR across the total cohort of 2052 patients, and just 65 of the 724 (9%) patients in the post‐mastectomy subgroup underwent BR, significantly lower than state averages. Significant relationships were found between age and reconstruction rates (*p* < 0.001), with younger patients (< 55 years old) more likely to opt for BR. Logistic regression confirmed that increased age and rurality both significantly affected the likelihood of undergoing reconstruction.

**Conclusion:**

Women aged < 55 years old and those residing in metropolitan areas showed a higher likelihood of opting for BR, highlighting the influence of age and rural residency on access to reconstruction services. These findings emphasise the need for targeted interventions to enhance BR access, particularly for older and rural patients.

## 1. Introduction

Breast cancer is the most common malignancy affecting women worldwide, with more than 2.3 million new cases and 685,000 deaths reported in 2020 [1]. Australia and New Zealand have the highest incidence rates globally (≈ 80 per 100,000), with 23,277 new cases and 3792 deaths recorded in 2020 [1]. The mean age at diagnosis in Australia in 2020 was 61 years [2]. A breast cancer diagnosis substantially affects physical function, psychological wellbeing and quality of life [3]. Surgical management—including lumpectomy or mastectomy, with or without adjuvant radiotherapy—remains a fundamental component of treatment [4].

Following mastectomy, breast reconstruction (BR) should be discussed with all eligible patients to support informed decision‐making regarding reconstructive surgery. International and national guidelines—including those from The National Institute for Health and Cancer Excellence (NICE), the National Comprehensive Cancer Network (NCCN) and Australian Specialty and Health‐Department Bodies—recommend that BR options (immediate and delayed) be routinely offered or discussed with patients undergoing mastectomy, irrespective of local service availability [5, 6]. Although BR is widely endorsed as part of contemporary best practice, uptake remains variable and is influenced by patient preference and system‐level access to reconstructive services [5–9].

Globally, studies have found that approximately 50% of eligible women would elect for reconstruction if they were adequately informed and offered the option [9–11]. Despite this, the national average of BR in Australia is reportedly only 29% [5, 10]. Most reconstructions occur in metropolitan populations, which represent ≈ 79% of national cases [2]. The 2023 Reporting for Better Cancer Outcomes from NSW [2] shows BR rates of 35%–45% in metropolitan areas but only 5%–17% in rural and regional settings, revealing a striking disparity in Australia. Metropolitan populations are defined as those residing in major cities of Australia. Rurality often denotes geographic remoteness rather than suburban or peri‐urban distance in Australia, with large regional populations often residing several hundred kilometres from the nearest tertiary hospital, sparse transport infrastructure and low specialist workforce density, creating major structural barriers to timely access to complex services such as BR.

Regional and rural populations comprise all areas outside major cities, classified as inner regional, outer regional, remote or very remote under the Australian Statistical Geography Standard (ASGS) or corresponding Modified Monash Model (MM2–MM7). Existing studies on these observed disparities are limited and report inconsistent findings regarding reasons.

These disparities are likely explained by a multifactorial interplay of access to specialist services and facilities, geographical location, referral patterns, comorbidities and personal preference [12].

There is a need for quantitative research in this area to address the discrepancies between rural and metropolitan patients accessing BR post‐mastectomy. The primary aim of this study is to assist in further identifying key contributors to lower rates of BR in rural populations. Additionally, it aims to quantify demographic and geographic factors associated with reduced access to reconstruction to inform service provision, targeted interventions and potential barriers to accessing reconstructive services and counselling. Furthermore, it aims to guide future research incorporating patient‐reported outcomes such as informed decision‐making processes and health‐related quality of life (HRQoL) and functional measures, particularly in regional and rural areas.

## 2. Methods

### 2.1. Study Design and Literature Review

A comprehensive literature review was conducted prior to data extraction to inform study design and identify gaps in knowledge pertaining to rural–metropolitan disparities in BR.

### 2.2. Setting

A multi‐centre, retrospective observational cohort study was performed. It comprised patients within Illawarra Shoalhaven Local Health District (ISLHD) who underwent management of breast cancer including an oncologic resection between 2012 and 2022. The ISLHD is a coastal health district in New South Wales, Australia, immediately south of Sydney, spanning a narrow coastal plain.

It covers the Wollongong, Shellharbour, Kiama and Shoalhaven local government areas (LGAs), with a land area of approximately 5,687 km^2^. Based on the Australian Bureau of Statistics estimated resident populations for these LGA’s, the ISLHD catchment population was 426,889 people in 2022.

This location was selected because it services a geographically diverse population ranging from metropolitan areas (classified as category 1 on the Modified Monash model (MM1)) to small rural towns (classified as category 5 (MM5)).

The ISLHD has a large tertiary hospital within 90 min’ drive of a rural/regional town with another smaller, secondary hospital. Both sites are governed by the same LHD and have the same access to investigation modalities, adjuvant therapies and multidisciplinary team (MDT) involvement. Access to reconstructive surgical care is available across two main public operating hospitals, with 22 operating theatres between the two and two private hospitals. Both breast and endocrine surgeries (general surgery subspeciality) and plastic surgery are available services within the district at both major district public hospitals including Wollongong and Shoalhaven. Breast cancer care is supported by district‐wide MDT involvement and breast care nursing services across the Illawarra and Shoalhaven cancer centres. Shoalhaven District Memorial Hospital provides plastic surgery services and receives clinical outreach support from Wollongong Hospital, reflecting a hub‐and‐spoke model of specialist access.

### 2.3. Data

Raw data were exported from the ISLHD SurgiNet data management system and imported into a secure file with restricted access and sharing privileges. These records were then correlated with medical and radiation oncology data held within the ISLHD’s MOSAIQ oncology information system in addition to the NSW cancer registry.

Patients were eligible for inclusion in the study if they met all of the following criteria: they were female, > 18 years of age, diagnosed with primary breast cancer (classified as per ICD‐10 codes C50.0—C50.9) between 2012 and 2022 and subsequently had a lumpectomy and/or mastectomy within ISLHD. There were 2052 eligible patients identified.

Data were deidentified following coding and data cleaning procedures. Clinical and demographical information for these patients is shown in Table 1. Some other relevant information was requested but was unable to be obtained from clinical files. This included the clinical indication for mastectomy (instead of lumpectomy), surgical and peri‐operative complications, referral pathway, completeness of staging at time of diagnosis and rates of local or regional recurrence.

**TABLE 1:** Descriptive statistics—total cohort.

Variable	Category	No reconstruction *N*	Reconstruction *N* (%)	Total (%)
Age Group	< 25	1	1 (50)	2 (0.1)
	25–34	22	12 (35.3)	34 (1.7)
	35–44	97	26 (21.1)	123 (6)
	45–54	297	46 (13.4)	343 (16.7)
	55–64	435	28 (6)	463 (22.5)
	65–74	586	11 (1.8)	597 (29.1)
	75–84	384	3 (0.7)	387 (18.9)
	85+	103	0 (0)	103 (5.0)
	Total	1925	127 (6.2)	2052 (100)

Rurality	MM1	1146	86 (7)	1232 (60)
	MM2	113	11 (8.9)	124 (6)
	MM3	361	15 (4)	376 (18.3)
	MM4	187	10 (5)	197 (9.6)
	MM5	114	4 (3.4)	118 (5.4)
	Unknown	4	1	5 (0.2)
	Total	1925	127	2052 (100)

Language	Non–English speaking	163	2 (1.2)	165 (8)
	English Speaking	1762	125 (6.6)	1887 (92)
	Total	1925	127	2052 (100)

Diabetes Status	No Diabetes	1584	123 (7.2)	1707 (83.2)
	Diabetes	341	4 (1.2)	345 (16.8)
	Total	1925	127	2052 (100)

Three subgroups of patients relating to lumpectomy ± subsequent mastectomy were evaluated and categorised: (1) lumpectomy‐only, (2) mastectomy‐only and (3) mastectomy with prior lumpectomy. This is because in lumpectomy alone, reconstruction is usually not required. However, a small number of patients coded as lumpectomy‐only were recorded as undergoing reconstruction, which may reflect oncoplastic or volume‐replacement procedures following breast‐conserving surgery, or limitations of administrative coding. Therefore, primary analyses focused on the mastectomy subgroups.

BR was defined as any surgical procedure coded within the SurgiNet data management system as a reconstructive breast operation performed following oncologic resection. This included oncoplastic techniques (such as shape and volume restoration), as well as implant‐based and autologous reconstruction procedures (such as local, regional or free flap reconstruction). However, the specific reconstructive technique (e.g., implant‐based versus autologous, or local/regional versus free flap) was not consistently available and could not be reliably disaggregated. Therefore, reconstruction was analysed as a binary outcome (reconstruction versus no reconstruction), including oncoplastic techniques post‐lumpectomy‐only.

Categorical variables were used for statistical evaluation. Age (coded into age‐group deciles spanning 10 years per group from < 25 to > 85), comorbidities, language (coded dichotomously as English‐speaking versus English as a second language), which was based on recorded primary language in clinical records, and all‐cause mortality for inclusion as present or not present. The time from the date of initial surgery to the date of death (where present) was calculated to evaluate 5‐year‐survival rates for surgery dates between 2012 and 2017. This allowed for a complete 5‐year survival interval to pass and be captured within our dataset. Rurality was coded via patient postcode and suburb at the time of treatment, which was categorised as MM1–MM5 according to the modified Monash Model of rural classification.

### 2.4. Statistical Analysis

Descriptive statistics were used to describe the cohort and all subgroups. Univariate chi‐squared analysis was used to evaluate the relationships between age, comorbidities, rurality and language with reconstruction rates in isolation. Multivariable logistic regression analysis with pairwise comparisons was used to evaluate the combined influence of the above variables with reconstruction rates in each subgroup.

To determine whether specific subgroups differed in their likelihood of undergoing BR, post hoc pairwise comparisons were conducted for the categorical variables of age and rurality. Odds ratios with 95% confidence intervals were used to quantify the strength and direction of these associations, allowing comparison of the relative likelihood of reconstruction between groups (e.g., between different age categories or metropolitan versus regional patients). This approach enabled clinically interpretable estimates of how demographic and geographic factors influenced the probability of reconstruction, beyond simple descriptive percentages. All statistical analyses were performed using Jamovi (version 2.3).

### 2.5. Statement of Ethics

This study was approved by the Greater Western Human Research Ethics Committee (2024/ETH00414). All data were handled in compliance with the NSW Health data‐governance policy.

## 3. Results

### 3.1. Cohort Characteristic

There were 2052 female patients aged 23–94 years (mean 65 ± 13.2) who met the inclusion criteria. Baseline demographics are summarised in Table 1. Approximately half of the cohort (52%) were aged 55–74 years. English was the first language in 92% of patients. A diagnosis of diabetes (either type 1 (T1DM) or type 2 (T2DM)) was present in 16.8% of patients. By rurality (Modified Monash Model, MMM), 60% resided in MM1 (metropolitan Illawarra), 6% in MM2, 18% in MM3, 10% in MM4, 5% in MM5 and the remaining 1% had no documented address.

Of the 2052 patients who underwent a surgical procedure for breast cancer, including lumpectomy ± mastectomy, 127 (6.2%) underwent some form of BR during the study period. Of these 127 BRs, 45 (35.4%) were immediate reconstructions, 73 (57.5%) were delayed BRs and 9 (7.1%) reconstructions were of unclear timing due to missing data pertaining to reconstruction in instances where reconstruction was performed out of area.

There were 1328 patients who had lumpectomy only, of whom 62 (4.7%) of these patients were coded as having undergone a breast reconstructive procedure, as per the operative coding definition described in methodology.

There were 724 patients who underwent either a mastectomy only or had a mastectomy following an initial lumpectomy; of these patients, 65 (9%) underwent a breast reconstructive procedure. The patients who underwent a mastectomy had a similar distribution of age and rurality compared to the overall study cohort (Table 2). Patients in the mastectomy ± prior lumpectomy subgroup in Table 2 refer to those who underwent an initial lumpectomy and within the specified timeframe were also noted to have had a subsequent mastectomy at any stage. The indications for the subsequent mastectomy (such as recurrence, incomplete margins, patient preference or new primary, for example) were not consistently captured in the administrative dataset when coding and so could not be further evaluated in this study.

**TABLE 2:** Descriptive statistics—mastectomy ± prior lumpectomy.

^Variable^	^Category^	^No reconstruction *N*^	^Reconstruction *N* (%)^	^Total (%)^
^Age Group^	^< 25^	^1^	^1 (50)^	^2 (0.3)^
	^25–34^	^10^	^8 (44.4)^	^18 (2.5)^
	^35–44^	^39^	^16 (29.1)^	^55 (7.6)^
	^45–54^	^105^	^23 (18)^	^128 (17.7)^
	^55–64^	^132^	^12 (8.3)^	^144 (19.9)^
	^65–74^	^153^	^5 (3.2)^	^158 (21.8)^
	^75–84^	^169^	^0 (0)^	^169 (23.3)^
	^85+^	^50^	^0 (0)^	^50 (6.9)^
	^Total^	^659^	^65 (9)^	^724 (100)^

^Rurality^	^MM1^	^344^	^47 (11.8)^	^391 (54)^
	^MM2^	^29^	^6 (17.1)^	^35 (4.8)^
	^MM3^	^152^	^4 (2.6)^	^156 (21.5)^
	^MM4^	^81^	^5 (5.9)^	^85 (11.9)^
	^MM5^	^51^	^3 (5.6)^	^54 (7.5)^
	^Unknown^	^2^	^0 (0)^	^2 (0.3)^
	^Total^	^659^	^65^	^724 (100)^

^Language^	^Non−English speaking^	^54^	^0 (0)^	^54 (7.5)^
	^English Speaking^	^605^	^63 (9.4)^	^668 (92.3)^
	^Total^	^659^	^63^	^721 (99.6)^

^Diabetes Status^	^No Diabetes^	^529^	^63 (10.6)^	^592 (81.8)^
	^Diabetes^	^130^	^2 (1.5)^	^132 (18.2)^
	^Total^	^659^	^65^	^724 (100)^

### 3.2. Distribution by Age and Rurality

The distribution of patients by age group and MMM classification demonstrated a progressive rise in rural residency with advancing age. Chi‐squared analysis tested whether there was an association between patient factors and reconstructive procedures (Table 3), as described below.

**TABLE 3:** Chi‐squared tests of association between patient variables and breast reconstruction.

Subgroup	Parameter vs. reconstruction	*X* ^2^	DF	*p* value∗
Total Cohort (*N* = 2052)	Age	180	7	**< 0.001**
	Rurality	9.65	5	0.086
	Language	7.65	1	**0.006**
	Diabetes	18.1	1	**< 0.001**

Mastectomy only plus mastectomy with prior lumpectomy (*N* = 724)	Age	99.9	7	**< 0.001**
	Rurality	17.2	5	**0.004**
	Language	2.17	1	0.141
	Diabetes	11	1	**< 0.001**

^∗^ *p* < 0.05 was considered statistically significant (bold values indicate significance).

#### 3.2.1. Age

Age showed a highly significant relationship with reconstruction (*p* < 0.001). Notably, patients aged 55–64 and 65–74 years had reconstruction rates of 6% and 1.8%, respectively, compared with 35% in the 25–34‐year‐old group. When age was dichotomised (< 55 vs. ≥ 55 years), patients older than 55 years had an odds ratio (OR) of 0.14 (*p* < 0.001), indicating an 86% lower likelihood of reconstruction compared to younger patients less than 55 years old.

#### 3.2.2. Rurality

Rurality significantly influenced BR rates in the mastectomy subgroup (*p* = 0.004) (Table 3) but did not significantly influence BR rates in the total cohort (*p* = 0.086). Patients residing in MM3 locations were 77% less likely to undergo BR than those residing in MM1 locations (OR 0.23, *p* = 0.008). Reconstruction rates declined with increasing MM category—declining from 12.1% in MM1 to ≤ 6% in MM3–MM5. This corresponded to a 70%–80% lower odds of reconstruction with increasing rurality.

#### 3.2.3. Language

English as a second language was significantly associated with decreased BR rates in the total cohort (*p* = 0.006) but not within the mastectomy subgroup (*p* = 0.141).

#### 3.2.4. Multivariable Logistic Regression

Logistic regression modelling (Tables 4 and 5) confirmed a consistent inverse association between age and reconstruction in the total cohort and mastectomy subgroups. As age increased, reconstruction likelihood declined steeply. Compared with women aged 25–34 years, those aged 55–64 had an adjusted OR of 0.12 (*p* < 0.001) and those aged 65–74 had an adjusted OR of 0.04 (*p* < 0.001)—representing an 88%–96% reduction in odds of reconstruction.

**TABLE 4:** Multivariable logistic regression analysis—total cohort.

Predictor	Pairwise comparison	*β* (log‐odds)	*Z*	*p* value∗	OR (95% CI)
*Intercept Rurality*	—	−1.872	−2.340	0.019	0.154
MM1	MM2	0.459	1.285	0.199	1.583
	MM3	−0.549	−1.827	0.068	0.578
	MM4	−0.188	−0.524	0.600	0.828
	MM5	−0.554	−1.036	0.300	0.575

MM2	MM3	−1.008	−2.314	**0.021**	0.365
	MM4	−0.647	−1.366	0.172	0.523
	MM5	−1.013	−1.639	0.101	0.363

MM3	MM4	0.360	0.824	0.410	1.433
	MM5	−0.005	−0.009	0.993	0.995

MM4	MM5	−0.365	−0.589	0.556	0.694

*Age Group:*					
25–34	< 25	0.411	0.281	0.778	1.509
	35–44	−0.722	−1.689	0.091	0.486
	45–54	−1.336	−3.340	**< 0.001**	0.263
	55–64	−2.137	−5.118	**< 0.001**	0.118
	65–74	−3.204	−6.681	**< 0.001**	0.041
	75–84	−4.092	−5.940	**< 0.001**	0.017

35–44	45–54	−0.614	−2.212	**0.027**	0.541
	55–64	−1.414	−4.674	**< 0.001**	0.243
	65–74	−2.482	−6.441	**< 0.001**	0.084
	75–84	−3.370	−5.377	**< 0.001**	0.034

45–54	55–64	−0.800	−3.133	**0.002**	0.449
	65–74	−1.868	−5.341	**< 0.001**	0.155
	75–84	−2.756	−4.557	**< 0.001**	0.064

55–64	65–74	−1.067	−2.920	**0.004**	0.344
	75–84	−1.955	−3.186	**0.001**	0.142

65–74	75–84	−0.888	−1.353	0.176	0.412

Language	—	1.460	1.982	**0.047**	4.307

Diabetes	—	−0.945	−1.787	0.074	0.389

^∗^ *p* < 0.05 was considered statistically significant (bold values indicate significance).

**TABLE 5:** Multivariable logistic regression analysis—mastectomy ± prior lumpectomy.

Predictor	Pairwise comparison	*β* (log‐odds)	*Z*	*p* value∗	OR (95% CI)
*Intercept Rurality*	—	−0.692	−0.765	0.444	0.501
MM1	MM2	0.844	1.544	0.123	2.326
	MM3	−1.459	−3.645	**0.008**	0.232
	MM4	−0.479	−0.914	0.361	0.619
	MM5	−0.374	−0.513	0.568	0.688

MM2	MM3	−2.303	−3.145	**0.002**	0.100
	MM4	−1.323	−1.876	0.061	0.266
	MM5	−1.217	−1.515	0.130	0.296

MM3	MM4	0.980	1.367	0.172	2.665
	MM5	1.086	1.328	0.184	2.961

MM4	MM5	0.106	0.133	0.894	1.111

*Age Group:*					
25–34	35–44	−0.735	−1.265	0.206	0.480
	45–54	−1.305	−2.372	**0.018**	0.271
	55–64	−1.902	−2.580	**0.001**	0.149
	65–74	−2.640	−3.842	**< 0.001**	0.071
	75–84	—	—	—	—

35–44	45–54	−0.571	−1.444	0.149	0.565
	55–64	−1.168	−2.580	**0.010**	0.311
	65–74	−1.905	−3.306	**< 0.001**	0.149
	75.84	—	—	—	—

45–54	55–64	−0.597	−1.509	0.131	0.550
	65–74	−1.334	−2.508	**0.012**	0.263
	75–84	—	—	—	—

55–64	65–74	−0.737	−1.318	0.187	0.478
	75–84	—	—	—	—

65–74	75–84	—	—	—	—

Language	—	0.799	−1.225	0.322	2.222

Diabetes	—	−0.949	−1.225	0.220	0.387

^∗^ *p* < 0.05 was considered statistically significant (bold values indicate significance).

Pairwise comparisons highlighted progressive declines in BR with age: 55–64 vs. 35–44 years (OR 0.24, *p* < 0.001), 65–74 vs. 35–44 (OR 0.08, *p* < 0.001), and 75–84 vs. 35–44 (OR 0.03, *p* < 0.001). These correspond to reductions of 76%–97% in odds across successively older groups.

#### 3.2.5. Mastectomy ± Lumpectomy Subgroup

BR was significantly less likely with increasing age and rurality. Compared with patients aged 25–34 years, those aged 55–64 (OR 0.15; *p* = 0.001) and 65–74 (OR 0.07; *p* < 0.001) were far less likely to undergo reconstruction. Patients in MM3 were also significantly less likely to have reconstruction than those in MM1 (OR 0.23; *p* = 0.008) and MM2 (OR 0.10; *p* = 0.002). Pairwise comparisons confirmed a progressive decline with advancing age, including 55–64 vs 35–44 (OR 0.31; *p* = 0.010) and 65–74 vs. 45–54 (OR 0.26; *p* = 0.012). Overall, increasing age and greater rurality were associated with substantially reduced likelihood of BR.

#### 3.2.6. Summary

Increasing age and greater rurality were the strongest negative predictors of reconstruction. Patients aged ≥ 55 years and those residing in MM3 or more remote areas were 75%–95% less likely to undergo BR compared with younger metropolitan patients. These patterns remained consistent across univariate and adjusted models, suggesting persistent age‐ and geography‐related disparities in reconstructive access within the Illawarra Shoalhaven region.

## 4. Discussion

The findings highlight substantial disparities in BR rates when compared with national and regional averages. The rate of BR was just 9% following any mastectomy, and only 6% following any kind of oncologic resection (lumpectomy or mastectomy), acknowledging that a small number of reconstruction codes were recorded following lumpectomy alone, likely representing oncoplastic techniques rather than reconstruction by definition [13]. These rates are concerning irrespectively. The Australian national averages are estimated to be around 30% overall and up to 45% in metropolitan areas [2]. Age was shown to be a key determinant of BR, with younger women—especially those under 45—being significantly more likely to opt for reconstruction compared to older age groups. This is an expected result and can be explained as reflecting the relative importance of body image, self‐esteem and longer‐term post‐surgery quality of life for younger patients [14]. However, given that younger adults tend to live in more metropolitan locations, it may be too simplistic to think of this as simply an age‐related phenomenon.

This study confirms that as age increases, rural residency also disproportionately increases, which subsequently decreases access to available health services and patient engagement. This may mean that rurality is interconnected with patient age, compounding the effect of age on decreased BR rates in older patients. Despite patients aged > 55 years accounting for the largest proportion of breast cancer diagnoses, access to specialised BR services and adequate infrastructure remain more limited in regional and rural settings. In this context, infrastructure refers specifically to the availability of subspecialist breast and reconstructive surgeons, multidisciplinary breast units and perioperative resources required to support immediate or delayed reconstruction, which are disproportionately concentrated in metropolitan centres [15].

With nearly 50% of all patients between the age of 55 and 75 living outside of major metropolitan areas in this study, i.e., MM2–MM5, this is disconcerting. The results imply that geographical location, as defined by the Modified Monash Model, plays a critical role in determining access to BR services for these patients.

There may be other factors aside from rurality and age that contribute to disparity in BR rates. This could include perceived age‐related surgical risk, competing comorbidities and limited referral pathways and accessible services in more regional areas [l]. In addition, many older women prioritise management of chronic disease or perceive reconstruction as unnecessary or purely cosmetic, for example, and it has been hypothesised that this may be contributing [16].

Diabetes was included a priori as a comorbidity of interest because it has previously been a recognised determinant of perioperative risk and is associated with higher rates of surgical site infection. In BR specifically, evidence demonstrates increased overall and wound‐related complications (including infection, necrosis and implant/flap failure) in patients with diabetes, which may influence both perceived suitability for reconstruction and uptake [17, 18]. However, contemporary evidence indicates that BR is still safe and beneficial across age groups, improving body image, self‐esteem and quality of life without increasing complications relative to the risks associated with increasing age and comorbidities overall [19].

Furthermore, geographical inequities likely reflect shortages of reconstructive surgeons, limited perioperative infrastructure and delays in multidisciplinary consultation. Patients in rural areas often face long travel distances, extended wait times and fragmented postoperative care, reducing the feasibility of both immediate and delayed BR [20].

When fully informed of all available options regarding breast surgery and reconstruction, women are far more likely to elect reconstruction [21]. Previous studies show that comprehensive counselling increases uptake to BR intervention without added morbidity. Wong et al. reported that in a specialist centre where full disclosure of BR options was made the standard of care over a 3‐year study period, 41% of eligible women opted for immediate BR, versus national averages < 15% prior to the 3‐year study. They also reported increased HRQoL scores without increases in complication rates or delayed cancer care [21].

The association between advanced age, rural residency and reduced reconstruction rates may be more complex than it appears. The association may be at least partially explained by rurality limiting timely access not only to the necessary services and infrastructure for BR procedures but also to older patients (and their referring and treating doctors) perceptions and acceptability of BR overall [12]. Further evaluation in prospective qualitative studies should explore this dynamic in greater depth with patient‐reported outcome measures and perceptions of BR identified. A number of studies have explored women’s experiences and perceptions regarding BR, including qualitative analyses of patient‐reported support needs, decision‐making influences and reconstruction outcomes, which highlight the complexity of factors shaping women’s views on reconstruction and support the need for further in‐depth exploration in regional cohorts [22–24].

This study has several limitations. Although conducted across multiple sites, data were derived from a single health district, limiting generalisability to broader populations.

Although language was statistically associated with reconstruction in the total cohort, the number of non–English‐speaking patients was small (8%), and this variable was not considered further due to limited power and potential confounding, although it may be an area of interest in future research.

The analysis was constrained by unavailable variables, including clinical stage, tumour characteristics, surgical complications, recurrence rates, adjuvant therapies and referral pathways. These missing data reduce analytical granularity and the ability to fully adjust for confounding factors. Additionally, reliance on retrospective, historical data may not reflect current healthcare services or advances in reconstructive techniques and perioperative care.

While this retrospective study effectively demonstrates significant disparities in BR based on age and rurality, it lacks the qualitative depth required to explore underlying motivations, perceptions and barriers to reconstruction—particularly among older and rural patients. Without qualitative insights, it remains unclear how stigma, misinformation or provider bias may influence patient decision‐making.

In light of these findings and limitations, we plan to conduct future research with prospective, mixed‐methods designs incorporating qualitative interviews and patient‐reported outcome measures to capture nuanced experiences and motivations behind BR decisions. Expanding to multi‐centre and longitudinal studies across diverse regions will improve external validity and enable assessment of temporal trends as surgical practices and healthcare accessibility evolve.

Further work should also integrate additional clinical variables—tumour stage, adjuvant therapy, recurrence, type of oncoplastic or reconstruction technique and referral pathways—to strengthen analytical robustness and transparency. Investigating cultural attitudes, physician communication and systemic barriers—especially within rural and ageing populations—will also provide essential context for disparities observed in this study.

Ultimately, incorporating both patient and provider perspectives will inform strategies that promote equitable access to BR, improve shared decision‐making and address modifiable barriers related to geography, age and perception of surgical risk. These insights will guide policy and service delivery frameworks aimed at ensuring that all patients, regardless of age or location, can make informed choices regarding BR.

## Author Contributions

Steven J. Craig conceptualised the study, performed formal analysis, performed investigation, proposed methodology, performed supervision, contributed to validation and reviewed and edited the article. Samuel M. Jansson contributed to data curation, performed formal analysis, performed investigation, proposed methodology, provided resources and software, performed project administration, wrote the original manuscript draft, reviewed and edited the article and contributed to validation. Calyb J. Austin contributed to data curation, performed investigation and reviewed and edited the article. Mingchun Liu contributed to data curation, performed investigation and reviewed and edited the article.

## Funding

No funding was received for this manuscript.

Open access publishing was facilitated by the University of Wollongong, as part of the Wiley—University of Wollongong agreement via the Council of Australasian University Librarians.

## Disclosure

This research was conducted as part of the requirements of the Master of Surgery in the Discipline of Surgery, Sydney Medical School, Faculty of Medicine and Health, University of Sydney.

## Conflicts of Interest

The authors declare no conflicts of interest.

## Data Availability

The data that support the findings of this study are available on request from the corresponding author. The data are not publicly available due to privacy or ethical restrictions.
